# *APOE*, *CETP* and *LPL* genes show strong association with lipid levels in Greek children^☆^

**DOI:** 10.1016/j.numecd.2009.02.005

**Published:** 2010-01

**Authors:** M.C. Smart, G. Dedoussis, E. Louizou, M. Yannakoulia, F. Drenos, C. Papoutsakis, N. Maniatis, S.E. Humphries, P.J. Talmud

**Affiliations:** a Division of Cardiovascular Genetics, British Heart Foundation Laboratories, Department of Medicine, Royal Free and UCL Medical School, London, UK; b Department of Nutrition and Dietetics, Harokopio University, Athens, Greece; c Department of Biology, University College London, London, UK

**Keywords:** Obesity, Apolipoproteins, Single nucleotide polymorphisms, Genetic variants, Lipids, T2D, type 2 diabetes, GENDAI, Gene–Diet Attica Investigation on childhood obesity, APO, apolipoprotein, TG, triglyceride, LPL, lipoprotein lipase, CETP, cholesterol ester transfer protein, BMI, body mass index, SNPs, single nucleotide polymorphisms, HWE, Hardy–Weinberg equilibrium, TC, total cholesterol, LDL-C, low-density lipoprotein cholesterol, HDL-C, high-density lipoprotein cholesterol, PCA, principal component analysis, PC1, first principal component, PC2, second principal component, MAF, minor allele frequency, GWAS, genome wide association studies, TGRL, triglyceride rich lipoproteins, NS, not statistically significant, CI, confidence intervals, IQR, inter quartile range, AA, amino acid

## Abstract

**Background and aims:**

Studies have consistently demonstrated that variants in a number of candidate genes are significant determinants of lipid levels in adults. However, few studies have investigated the impact of these variants in children. Therefore, in the present investigation we examined the influence of ten common variants in the genes for lipoprotein lipase (*LPL* – S447X), cholesterol ester transfer protein (*CETP* – *Taq*1B) apolipoprotein (*APO*) E (ɛ2, ɛ3, ɛ4), *APOA5* (−1131C > T and S19W), *APOA4* (S347T) and *APOC3* (−482C > T; 1100C > T and 3238G > C) on lipoprotein levels children from the Gene–Diet Attica Investigation on childhood obesity (GENDAI).

**Methods and results:**

The ten variants selected were genotyped in 882 Greek children, mean age: 11.2 ± 0.7 years (418 females and 464 males). Genotypes were assessed using *Taq*Man technology. Significantly higher total cholesterol (TC) (*p* = 0.0001) and low-density lipoprotein cholesterol (LDL-C) (*p* < 0.0001) were observed in *APOE* ɛ4 carriers compared to ɛ3/ɛ3 homozygotes and ɛ2 carriers. The association of *APOE* genotype with TC and high-density lipoprotein cholesterol (HDL-C) ratio (*p* = 0.0008) was further modulated by body mass index. Carriers of the *CETP Taq*IB B2 allele had significantly higher HDL-C (*p* < 0.0001) and significantly lower TC: HDL-C ratio (*p* < 0.0001) compared to B1/B1 individuals. No significant associations were observed between *APOA4*, *APOA5* and *APOC3* variants and serum lipids.

**Conclusion:**

This study demonstrates that these common variants are associated with lipid levels in this healthy paediatric cohort, suggesting that even in these young children there may be potential in predicting their lifelong exposure to an adverse lipid profile.

## Introduction

In parallel with the increase in adult obesity, childhood obesity is a rapidly growing health problem worldwide [1]. Obesity in childhood is linked to many serious health complications usually seen in adulthood [2]. Co-morbidities include elevated blood pressure, increased prevalence of factors associated with type 2 diabetes (T2D) and lipid abnormalities [1]. The Gene–Diet Attica Investigation on childhood obesity (GENDAI) [3] was established to specifically explore the contribution of genetics and environmental factors in the development of childhood obesity. The GENDAI cohort consists of young children of both sexes attending school in the area of Attica, Greece. Preliminary assessment provided the impetus for a more detailed study of the metabolic syndrome phenotype in the GENDAI cohort with particular focus on the genetic contribution to inter-individual variation in plasma lipids in the young and the potential modulation of these genetic associations by environmental influences.

Genetic factors are considered to be important determinants of plasma lipoprotein levels in adults; however, the role of genetics in determining plasma lipoproteins in children and adolescents is less clear. The apolipoprotein (*APO*) cluster including *APOA5*, *APOAIV*, *APOC3* and *APOA1* on chromosome 11 is associated with variation in triglycerides (TG) in both children and adults and across a range of ethnic groups [4,5]. Variation in the *APOE*, lipoprotein lipase (*LPL*) and cholesterol ester transfer protein (*CETP*) genes has been consistently associated with variation in lipid levels in adults [6,7]. However, genetic variation in these gene loci explain only a modest proportion of inter-individual variability in fasting lipid levels [8]. We genotyped the GENDAI cohort for ten variants in the *APOE*, *LPL*, *CETP* genes and the *APOA5/A4/C3* cluster, to examine if the reported effects could be replicated in children and assess if these associations could be further modulated by body mass index (BMI).

## Methods

### Study population

Participants were drawn from the children recruited in the GENDAI study. Briefly, a random sample of 2492 children attending school in the Attica region in Greece were invited to join the study. A total of 1138 children were recruited from November 2005 to June 2006. Due to the heterogeneity in allele frequencies between Greek and non-Greek Caucasians, only children of Greek nationality, mean age: 11.2 ± 0.7 years (*n* = 882; 418 males and 464 females), were included in the present study. Details of recruitment and data collection have been previously described [3]. All persons gave their informed consent prior to inclusion in the study. The study was approved by the Institutional Review Board of Harokopio University and the Greek Ministry of Education [3].

## Experimental procedures

### Single nucleotide polymorphism selection and genotyping

A salting-out procedure [9] was used to isolate DNA samples from whole blood. Ten single nucleotide polymorphisms (SNPs) in six candidate genes; *LPL* S447X (rs328), *CETP Taq*1B (rs708272), *APOE* (rs7412, rs429358), *APOA5* −1131C > T (rs662799) and S19W (rs3135506), *APOA4* S347T (rs675) and *APOC3* −482C > T (rs2854117), 1100C > T (rs4520) and 3238C > G (rs5128) were genotyped using *Taq*Man technology (Applied Biosciences, ABI, Warrington UK). Reactions were performed on 384-well microplates and analysed using ABI *Taq*Man 7900HT software. Primers and MGB probes are available upon request.

## Statistical methods

Hardy–Weinberg equilibrium (HWE) was examined by chi-square goodness of fit test. A *p* value of <0.05 was taken as deviation from HWE. Plasma levels of insulin, TG, total cholesterol (TC), low-density lipoprotein cholesterol (LDL-C) and high-density lipoprotein cholesterol (HDL-C) and all anthropometric measures were natural log-transformed. For association studies a *p* value of <0.01 was taken as statistically significant. Setting a threshold of significance was the chosen method above Bonferroni corrections, since the candidate genes studied had been selected for based on a *priori* hypothesis and biological plausibility. A *p* value of <0.05 was taken as statistically significant in Principal Component Analysis (PCA). The majority of statistical analyses were performed using Intercooled Stata 8.2 for Windows (StataCorp LP, Texas, USA). Haplotype association analysis was carried out using Thesias [10]. PCA was carried out using SAS (SAS Institute Inc., Cary, NC). Throughout this study we used the first principal component (PC1) and the second principal component (PC2) because both components explained more than 80% of the variation and their eigenvalues were greater than the average eigenvalue [11].

## Results

Subjects were classified as obese, overweight and non-overweight according to the International Obesity Task Force [12]. The non-overweight group includes normal weight (*n* = 533) and underweight (*n* = 2) children and for the purposes of this study has been called the normal weight group (*n* = 535). Table 1 summarises the baseline characteristics of a selection of lipid and anthropometric measures, comparing the overweight and obese children (*n* = 343) in the study to their normal weight counterparts (*n* = 535). Measures of blood pressure, insulin, TG, height and insulin resistance were significantly higher (*p* < 0.0001) and HDL-C significantly lower (*p* < 0.0001) in the overweight and obese group compared to their normal weight counterparts. The mean BMI of the mothers and fathers of the children was 24.6 ± 4.8 and 27.2 ± 3.6, respectively. Children whose parents were both overweight (BMI ≥ 25) had a BMI 2.2 units larger than children whose parents were both of a normal weight (BMI < 25) (*p* < 0.00001) (Appendices Table 1a). Children of parents who were hypercholesterolemic (Cholesterol >240 mg/dl) had significantly higher cholesterol levels compared to children of parents who's cholesterol levels were normal (*p* < 0.00001) and the same observation was observed between parents and their children with LDL levels (*p* = 0.003) (Appendices Table 1c and 1b, respectively).

### Genotype frequencies

There were no allele frequency differences between boys and girls for any of the ten variants (data not shown). The genotype and minor allele frequency (MAF) for the ten variants are shown in Table 2. The genotype distribution of *APOC3* 1100C > T deviated from those expected under HWE (*p* = 0.02). There was a borderline statistically significant difference in allele frequency distribution of the *LPL* S447X variant between normal weight children and their overweight and obese counterparts, MAF 0.14 (95% confidence intervals (CI) 0.11, 0.16) vs. 0.11 (95% CI 0.08, 0.14) (*p* = 0.02).

### Association with quantitative traits

Significant data for lipid and anthropometric variables, according to genotype, are presented in Table 3. *APOE* genotype, defined by two variant sites at residues 112 and 158 creating the ɛ2, ɛ3 and ɛ4 alleles, was significantly associated with TC (*p* = 0.0001) with ɛ2 carriers having TC plasma levels 11.3% lower than ɛ3/ɛ3 subjects (*p* < 0.001) and ɛ4 carriers with 1.3% higher TC plasma levels than ɛ3/ɛ3 subjects (*p* = 0.522). *APOE* genotype was also significantly associated with LDL-C (*p* < 0.0001). ɛ2 carriers had LDL-C plasma levels that were 17.6% lower than ɛ3/ɛ3 subjects (*p* < 0.001) and ɛ4 carriers had a mean LDL-C plasma level that was 2.8% higher than ɛ3/ɛ3 subjects (*p* = 0.258). ɛ2 carriers were also observed with a significantly lower mean TC: HDL-C ratio of 3.26 (95% CI 3.1, 3.5) compared to 3.62 (95% CI 3.6, 3.7) and 3.81 (95% CI 3.7, 4.0) for ɛ3/ɛ3 and ɛ4 carriers, respectively (*p* = 0.0008). The *CETP Taq*1B variant was associated with significantly higher HDL-C (*p* < 0.0001) and lower TC: HDL-C ratio (*p* < 0.0001) in those who were carriers or homozygotes for the B2 allele.

The *LPL* S447X variant showed borderline significant association with weight (*p* = 0.02) with 447X homozygotes weighing almost 5 kg less than S447 homozygotes. Carriers of the *APOA5* 19W had 7.8% higher plasma TG levels compared to homozygotes for the common allele, but the association did not reach statistical significance (*p* = 0.038) (Appendices Table 2). Carriers of the *APOA5* −1131 rare T allele had 6.1% higher TG levels compared to CC homozygotes, but again this did not reach statistical significance (*p* = 0.048) (Appendices Table 2). No significant associations were observed between the *APOA4* T347S and *APOC3* variants and serum lipids. Common haplotypes within the *APOA5/A4/C3* cluster showed no significant effect on TG, TC HDL-C or LDL-C levels (Appendices Table 2).

### Principal component analysis

The plasma and anthropometric measures used for PCA were combined based on their correlation structures (Appendices Table 3). The first PCA included HDL-C, TC and LDL-C with PC1 explaining 74% of the variation and PC2 an additional 25% of the variation. *CETP Taq*1B and *APOE* were identified as having a significant effect in both PC1 (*p* < 0.01) and PC2 (*p* < 0.001) (Table 4a). The second PCA included weight, waist circumference, hip circumference, triceps and subscapular skin folds. PC1 alone explained 84% of the total variation, identifying *LPL* S447X as significant (*p* = 0.04) (Table 4b). The final PCA combined measures of TG, insulin and insulin resistance with PC1 explaining 72% of the variation and PC2 explaining an additional 27%. PC2 identified the two variants in *APOA5*, −1131C > T and S19W as having a significant effect (*p* = 0.015 and *p* = 0.039, respectively) (Table 4c).

### *APOE* variation interacts with BMI impacting on lipid measures

**Figure 1:**
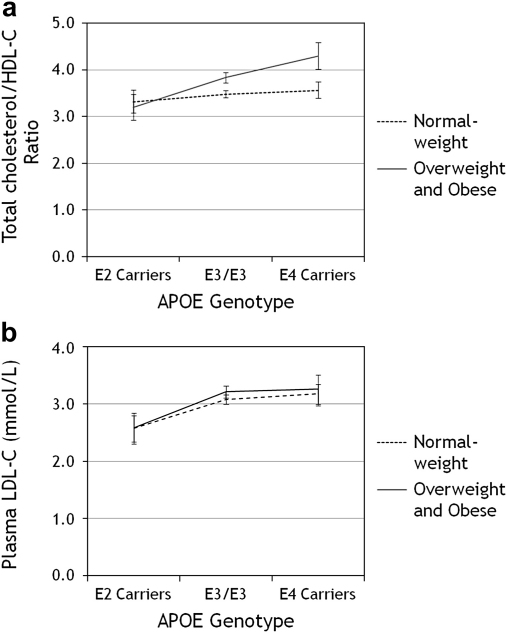
a) Interaction between *APOE* genotype and BMI category on total cholesterol/HDL-C ratio (*p* = 0.008). Normal weight children: ɛ3/ɛ3 = 195, ɛ2 carriers = 20, ɛ4 carriers = 43; Overweight and obese children: ɛ3/ɛ3 = 139, ɛ2 carriers = 14, ɛ4 carriers = 25. b) Interaction between *APOE* genotype and BMI category on plasma LDL-C (Not significant). Normal weight children: ɛ3/ɛ3 = 195, ɛ2 carriers = 19, ɛ4 carriers = 43; Overweight and obese children: ɛ3/ɛ3 = 136, ɛ2 carriers = 14, ɛ4 carriers = 2. APO, apolipoprotein; BMI, body mass index; HDL-C, high-density lipoprotein cholesterol, and LDL-C, low-density lipoprotein cholesterol.

An interaction of APOE and BMI (dichotomised into Normal weight and Overweight/Obese) was impacting on the TC: HDL-C ratio in this young cohort (*p* = 0.008) was identified (Fig. 1a) and HDL-C levels (*p* = 0.01) (data not shown). ɛ4 carriers in the overweight and obese category had a poorer TC: HDL-C ratio of 4.3 (95% CI 4.0, 4.6) compared to ɛ2 carriers with a ratio of 3.2 (95% CI 2.9, 3.5). Children in the normal weight category had a TC: HDL-C ratio which was unaffected by *APOE* genotype. ɛ4 carriers had a mean ratio of 3.6 (95% CI 3.4, 3.8), ɛ3/ɛ3 3.5 (95% CI 3.4, 3.6) and ɛ2 carriers 3.3 (95% CI 3.1, 3.6). There was no interaction between BMI and APOE genotype on plasma LDL-C (Fig. 1b); TG or TC levels (data not shown). Gene–diet interactions examining all variants with a number of dietary measures were investigated, but there were no significant results (data not shown).

## Discussion

The results from this study demonstrate in this dataset of healthy Greek children, the significant association of candidate gene variants with baseline anthropometric and lipid measures. More than a third of the children in this cohort were overweight with obesity reaching a level of 9.5%. This is comparable to a study in central Greece assessing 11-year-olds weight status where a total of 30.3% were reported to be overweight and 6.7% obese [13]. The fact that parental BMI was positively associated with their child's BMI highlights the importance of family history and environment in the development of obesity. The overweight and obese children in the current study had significantly higher arterial blood pressure, lower HDL-C levels, higher TG and increased insulin compared to their the normal weight counterparts. Higher Tanner scores and heights of the overweight and obese group also suggest earlier onset of puberty, which is often frequently observed in overweight and obese children [14]. Any interaction between sexual maturity and effects of allelic variation on lipid levels that may have occurred could be accounted for in analyses from the Tanner measures.

Genetic factors are considered important determinants of plasma lipid levels in adults, demonstrated in several of the recent genome wide association studies (GWAS) in which a number of candidate genes have been confirmed [15,16]. The meta-analysis of 3 GWAS by Willer et al. (2008) identified strong associations with variants in *APOA5/A4/C3/A1* cluster, *APOE*, *CETP* and *LPL* influencing plasma lipid concentrations. Although a number of associations comparable to those seen in adults were confirmed in this study, the role of genetic factors in the heterogeneity of plasma lipid levels in children is less clear. Replication of these variants in cohorts of children is needed.

In GENDAI, *APOE* genotypes were associated with differences in TC and LDL-C plasma levels and the TC: HDL-C ratio. The LDL-C and TC lowering effect of the ɛ2 allele reported in the recent meta-analysis [17] was also observed in this cohort of young Greek children. Carriers of the ɛ4 allele had LDL-C and TC levels that were 19.9% and 12.2% higher than carriers of the ɛ2 allele and 2.8% and 1.3% higher than ɛ3/ɛ3 subjects. The results of a 21-year longitudinal study on changes in serum lipids in 1233 Finns followed from childhood to adulthood consistently observed the ɛ2 allele to be associated with lower LDL-C levels and the ɛ4 allele with higher TC and LDL-C levels (*p* < 0.001 for all associations) in childhood. The LDL-C-lowering effect of the ɛ2 allele was an association that was tracked through to adulthood, having a greater effect with increasing age (*p* = 0.039). The association of the *APOE* genotype with plasma TC and LDL-C has been reported in children as young as 3 years old [18]. The fact that differences in lipid levels cannot be detected in children at birth by the *APOE* genotype leads to the conclusion that lipid levels are influenced by genetic and environmental factors in a child's very first years of life [18].

*CETP* is involved primarily in the transfer of lipids in reverse cholesterol transport, exchanging cholesterol from HDL-C for TG from TG-rich lipoproteins (TGRL) [19], thus influencing the TG content of TGRL particles and playing a major role in the determination of HDL-C levels [20]. Meta-analysis of *CETP Taq*1B has consistently shown association with HDL-C levels [21]. The association of the B2 allele with higher HDL-C levels was observed in this study. Homozygotes for the B2 allele had approximately 10% higher mean HDL-C levels compared to the B1/B1 individuals, comparable to that seen in adults [22]. A highly significant association of the *Taq*1B variant with TC: HDL-C ratios in the cohort were also observed, highlighting the importance of this particular genotype and its effects on HDL-C levels from a young age. In a cohort of 257 Dutch prepubescent boys and girls (aged 6.7–8.1 years) the same association with the *Taq*1B variant was reported, but dependant on *APOE* genotype [23].

*LPL* is a key lipolytic enzyme that plays a crucial role in the catabolism of triglycerides in TG-rich particles and the S447X variant in exon 9 results in premature truncation [24]. The *LPL* 447X genotype has been consistently associated in adult populations with a beneficial lipid profile conferring a protective effect against myocardial infarction [25]. Children homozygous for the rare 447X allele had approximately 2% lower TG levels than children who were homozygous for the common allele, but this did not reach statistical significance. The borderline association of the 447X allele with lower weight is interesting considering the significant difference in MAF (*p* = 0.02) between the normal weight and overweight children (MAF 0.14 and 0.11, respectively).

Numerous studies have investigated the association of genetic variation in the *APOA5/A4/C3/A1* cluster on lipid levels in adults [5,26]. The TG raising effect of the *APOA5* S19W variant seen in adults was also observed in this cohort, but this did not reach statistical significance. Previous studies have shown significant associations of the *APOA5* −1131T > C promoter variant with TG levels [5], and although the association of this variant in the present study was not statistically significant, TG levels were 6.1% higher in children who were carriers of the rare allele. There was no significant association with any of the baseline lipid measures with the *APO4* and three *APOC3* variants examined. These findings corroborate with the data on the association of variants in the *APOC3* gene with lipid levels in children in the Columbia Biomarkers Study [27]. Although, trends were observed with the *APOC3* variants they did not reach statistical significance. In particular, carriers of the S2 allele of the *APOC3* Sst1 variant was associated with higher TG levels, which is consistent with the recently published AVENA Study [28]. The lack of association in the case of both the *APOA5* and *APOC3* variants was due to insufficient power to detect the modest effect size these variants were having on TG levels. PCA was adopted to increase the power of detecting such associations, but did not provide any information over and above the results from the regression analysis, but all associations were confirmed. As well as the association of these variants with lipid levels, it is of importance that the effect and influence of these variants on plasma apolipoprotein levels is also investigated. In the present study we unfortunately did not have these measures.

Increased levels of obesity have been demonstrated to amplify genetic effects. Even in these young children, BMI through an interaction with *APOE* was modulating and determining the lipid parameters of the TC: HDL-C ratio, with the less beneficial ratio being found among ɛ4 carriers than among ɛ3/ɛ3 or ɛ2 carriers. The *APOE* genotype had little influence on the TC: HDL-C ratio in children of a normal BMI. A similar association was seen in a cohort of 266 healthy men with *APOE* ɛ2, ɛ3, ɛ4 genotype and TC, LDL-C and insulin levels. Individuals who were ɛ4 carriers had significantly higher (*p* = 0.04) TC, LDL-C and insulin levels compared ɛ3/ɛ3 or ɛ2 carriers, an association which was enhanced in the ɛ4 carriers as BMI increased [29]. These data suggest that effects of *APOE* alleles on lipids levels are partly dependent on and modulated by environmental variables such as BMI.

Previous genetic studies have demonstrated that variants investigated in this study are significant determinants of serum lipid levels in adults. However, only a few studies have investigated the association of these variants in children. The effects in the GENDAI study are of similar magnitude to those observed in adults, suggesting that even in these young children there is potential in predicting their long-term exposure to an adverse lipid profile. Kathiresan et al. have developed a genotype score for use in CHD risk assessment [30]. Using 9 SNPs in genes that determining plasma LDL and HDL cholesterol levels, they reported that addition of a genotype score to a CHD risk algorithm improved risk reclassification, even after adjustment for baseline lipid levels. This result importantly suggested that lipid-associated SNPs may provide incremental information about an individuals' risk beyond a single lipid measure and furthermore, although individual SNPs exert only a modest affect on lipid variation, in combination they may have a substantial influence. The data from this present study suggest the influence of variants is exerted at a very young age, and thus reflecting a lifelong exposure.

## Figures and Tables

**Table 1:** Comparisons between normal weight and overweight/obese^a^ subjects.

Variable	Normal-weight	*n*	Overweight and obese	*n*	*p* value
Gender (male/female) (%male)	236/297 (44.1%)	535	180/163 (52.5%)	343	0.015^c^
Age (years)	11.2 ± 0.6^d^	534	11.1 ± 0.6^d^	340	NS^b^
Tanner genitals or breast score	2 (IQR 2–3)^e^	532	3 (IQR 2–3)^e^	338	0.06^c^
Tanner pubic hair score	2 (IQR 2–3)^e^	532	2 (IQR 2–3)^e^	338	NS^c^

Variable	Normal weight^f^	*n*	Overweight and obese^f^	*n*	*p* value^g^
Weight (kg)	39.1 (38.6, 39.6)	535	53.1 (52.2, 53.9)	343	<0.0001
Height (cm)	147.8 (147.2, 148.4)	535	150.8 (149.3, 150.0)	343	<0.0001
Waist circumference (cm)	63.5 (63.0, 64.0)	535	77.1 (76.1, 77.9)	342	<0.0001
Triceps skin fold (mm)	15.0 (14.6, 15.4)	535	25.7 (25.1, 26.4)	340	<0.0001
Subscapular skin fold (mm)	8.2 (8.0, 8.4)	535	16.0 (15.5, 16.6)	340	<0.0001
Body fatness % (Slaughter eq.)	21.8 (21.3, 22.3)	455	39.1 (37.9, 40.4)	270	<0.0001
Systolic blood pressure (mmHg)	117.2 (116.0, 118.5)	531	123.9 (122.2,125.5)	340	<0.0001
Diastolic blood pressure (mmHg)	72.8 (71.8, 73.9)	529	77.6 (76.2, 79.0)	340	<0.0001
Insulin (uIU/mL)	5.8 (5.6, 6.1)	513	8.8 (8.3, 9.3)	325	<0.0001
Glucose (mg/dL)	85.2 (84.4, 86.0)	525	84.6 (83.6, 85.6)	332	NS
Insulin resistance (HOMA^IR^_-IR_)	1.22 (1.16, 1.28)	512	1.84 (1.73, 1.96)	332	<0.0001
TG (mmol/L)	0.63 (0.61, 0.65)	526	0.75 (0.75, 0.81)	332	<0.0001
TC (mmol/L)	4.79 (4.72, 4.86)	526	4.81 (4.72, 4.89)	332	NS
HDL-C (mmol/L)	1.38 (1.36, 1.41)	526	1.27 (1.24, 1.29)	332	<0.0001
LDL-C (mmol/L)	3.07 (3.02, 3.13)	526	3.13 (3.06, 3.21)	332	NS
Birthweight (g)	3241.6 (2167.67, 4184.40)	467	3297.3 (2319.14, 4403.03)	305	NS

NS, not statistically significant; TG, total triglyceride; TC, total cholesterol; HDL-C, high-density lipoprotein cholesterol; LDL-C, low-density lipoprotein cholesterol; IQR, inter quartile range and CI, confidence intervals.

a Overweight and obesity were defined according to the International Obesity Task Force recommendations, using international reference values.

b Assessed by ANOVA.

c Assessed by χ^2^.

d Mean (±standard deviation).

e Median (IQR).

f Mean (95% CI).

g Assessed by linear regression, adjusted for Tanner status and gender.

**Table 2:** Allele and genotype frequencies of candidate gene variants in Greek children.

Gene	rs Number	AA and nucleotide change	Genotype	*n*	%	MAF (95% CI)
*CETP*	708272	Taq1B G > A	GG-B1/B1	252	35.44	0.4
GA-B1/B2	351	49.37	(0.37, 0.42)
AA-B2/B2	108	15.19	

*APOE*	429358	C112R	C112/C158-ɛ2	34	7.78	ɛ2 −0.09
7412	R158C	C112/R158-ɛ3	335	76.66	(0.07, 0.10)
		R112/R158-ɛ4	68	15.56	ɛ4 −0.14
					(0.10, 0.18)

*APOC3*	2854117	−482C > T	CC	304	48.41	0.31
CT	262	41.72	(0.28, 0.33)
TT	62	9.87	

*APOC3*	4520^a^	1100C > T	CC	321	50.63	0.28
CT	277	43.69	(0.25, 0.30)
TT	36	5.68	

*APOC3*	5128	Sst1	GG-S1S1	477	76.44	0.13
3238G > C	GC-S1S2	137	21.96	(0.11, 0.14)
	CC-S2S2	10	1.6	

*APOA4*	675	S347T	TT	352	57.8	0.24
AT	221	36.29	(0.22, 0.26)
AA	36	5.91	

*APOA5*	662799	−1131T > C	TT	556	84.5	0.08
TC	97	14.74	(0.07, 0.10)
CC	5	0.76	

*APOA5*	3135506	S19W	GG	582	90.23	0.05
GC	62	9.61	(0.04, 0.06)
CC	1	0.16	

*LPL*	328	S447X	CC	540	76.49	0.13
CG	154	21.81	(0.11, 0.14)
GG	12	1.7	

AA, amino acid; MAF, minor allele frequencie; CI, confidence intervals; APO, apolipoprotein; *LPL*, lipoprotein lipase and *CETP*, cholesterol ester transfer protein.

a Borderline deviation from Hardy–Weinberg Equilibrium (*p* = 0.02).

**Table 3:** Association of *APOE*, *CETP Taq*1B and *LPL* S447X with baseline plasma and anthropometric measures.

*APOE*	Genotype (n)	LDL-C (mmol/L)^a^	TC (mmol/L)^b^	TC: HDL-C Ratio^c^
Mean (95% CI)	Mean (95% CI)	Mean (95% CI)
	ɛ2 Carriers (33)	2.58 (2.41, 2.76)	4.27 (4.04, 4.49)	3.29 (3.11, 3.48)
	ɛ3/ɛ3 (332)	3.13 (3.07, 3.20)	4.82 (4.74, 4.90)	3.62 (3.56, 3.67)
	ɛ4 Carriers (67)	3.22 (3.07, 3.37)	4.88 (4.70, 5.07)	3.78 (3.62, 3.93)
	*p* value	<0.0001	0.0001	0.0008

*CETP TAQ1B*	Genotype (n)	HDL-C (mmol/L)^d^	TC: HDL-C ratio^c^
		Mean (95% CI)	Mean (95% CI)
	B1B1 (248)	1.27 (1.24, 1.30)	3.75 (3.67, 3.83)
	B1B2 (348)	1.37 (1.34, 1.40)	3.53 (3.47, 3.60)
	B2B2 (108)	1.40 (1.35, 1.46)	3.45 (3.35, 3.56)
	*p* value	<0.0001	<0.0001

*LPL S447X*	Genotype (n)	Weight (kg)^e^
		Mean (95% CI)
	SS (534)	44.25 (43.65, 44.87)
	SX (151)	43.30 (42.21, 44.40)
	XX (12)	39.42 (35.98, 43.20)
	*p* value	0.02

APO, apolipoprotein; LDL-C, low-density lipoprotein cholesterol; TC, total cholesterol; HDL-C, high-density lipoprotein cholesterol; CI, confidence intervals; TG, total triglyceride; *LPL*, lipoprotein lipase; *CETP*, cholesterol ester transfer protein and BMI, Body mass index. Only data with *p* values ≤0.02 has been presented. Tanner status was combined into one variable from the two Tanner measures to produce a mean Tanner score.

a Adjusted for height.

b Adjusted for height and gender.

c Adjusted for gender, mean Tanner score and BMI.

d Adjusted for height, gender and BMI.

e Adjusted for height, mean Tanner score and gender.

**Table 4:** Principal component analysis.

Variant	PC1 (74%)		PC2 (27%)	
*F* value	*p* value	*F* value	*p* value
*CETP Taq*1B	3.38	0.006	28.01	<0.001
*APOE*	8.21	0.004	13.05	<0.001

a) HDL-C – LDL-C – TC				

Variant	PC1 (84%)	
*F* value	*p* value
*LPL* S447X	4.24	0.04

b) Weight – Waist – Hip – Triceps and Subscapular skin folds		

Variant	PC1 (72%)		PC2 (25%)	
*F* value	*p* value	*F* value	*p* value
*APOA5* S19W	1.52	0.218	4.29	0.039
*APOA5* −1131C > T	0.07	0.798	5.93	0.015

c) TG – insulin – insulin resistance				

Principal component analysis was performed on three separate clusters. a) HDL-C, LDL-C and TC; b) weight, waist circumference, hip circumference, triceps skin fold and subscapular skin folds; c) TG, insulin and insulin resistance^HOMA^. The variance for the first and second principal components (PC1 and PC2) is provided; *F* and *p* values are provided for each of the significant variants. *LPL*, lipoprotein lipase; HDL-C, high-density lipoprotein cholesterol; LDL-C; low-density lipoprotein cholesterol; TC, total cholesterol; *CETP*, cholesterol ester transfer protein; APO, apolipoprotein and TG, total triglyceride.
