# Supplementary material for: APOE, CETP and LPL genes show strong association with lipid levels in Greek children
Source: Nutr Metab Cardiovasc Dis. 2010 Jan;20(1):26–33. doi: 10.1016/j.numecd.2009.02.005 (PMC2807029; doi:10.1016/j.numecd.2009.02.005)
Supplement: Supplementary file 1 [file mmc1.doc]

Appendices Table 1a. Association of parental BMI with offspring BMI.

|  | Offspring Mean BMI^a^ (95% CI) | n |
| --- | --- | --- |
| Both Parents BMI <25 | 18.96 (18.5, 19.43) | 158 |
| One Parent with BMI ≥25<30 | 19.54 (19.26, 19.84) | 443 |
| Both Parents BMI ≥30 | 21.21 (20.78, 21.65) | 229 |
|  | p<0.00001 |  |

^a^ assessed by linear regression, adjusted for Tanner status, height and gender.

Appendices Table 1b. Association of parental LDL-C with offspring LDL-C.

|  | Offspring Mean LDL-C^b^ (95% CI) | n |
| --- | --- | --- |
| Both Parents with Normal LDL-C Levels | 3.05 (3.02, 3.011) | 765 |
| One Parent with High LDL-C Levels | 3.30 (3.14, 3.44) | 74 |
| Both Parents with High LDL-C Levels | 3.61 (3.24, 4.03) | 14 |
|  | p=0.0003 |  |

^b^ adjusted for height. High LDL-C >130mg/dl

Appendices Table 1c. Association of parental TC with offspring TC.

|  | Offspring Mean TC^c^ (95% CI) | n |
| --- | --- | --- |
| Both Parents with Normal TC Levels | 4.73 (4.68, 4.78) | 684 |
| One Parent with High TC Levels | 5.02 (4.88, 5.13) | 145 |
| Both Parents with High TC Levels | 5.44 (5.18, 5.84) | 23 |
|  | p<0.00001 |  |

^c^ adjusted for height and gender. High Cholesterol >240mg/dl

BMI – Body mass index, CI – confidence intervals, LDL-C - low-density lipoprotein cholesterol, TC - total cholesterol.
