# Supplementary material for: APOE, CETP and LPL genes show strong association with lipid levels in Greek children
Source: Nutr Metab Cardiovasc Dis. 2010 Jan;20(1):26–33. doi: 10.1016/j.numecd.2009.02.005 (PMC2807029; doi:10.1016/j.numecd.2009.02.005)
Supplement: Supplementary file 2 [file mmc2.doc]

Appendices Table 2. Association of *APOC3*, *APOA5* and *APOA4* variants with baseline plasma measures.

| **Variant** | **Genotype (n)** | **TGs (mmol/L)**^a^ | **LDL-C (mmol/L)**^b^ | **TC (mmol/L)**^c^ | **HDL-C (mmol/L)^d^** | **TC: HDL-C Ratio**^e^ |
| --- | --- | --- | --- | --- | --- | --- |
|  |  | Mean (95% CI) | Mean (95% CI) | Mean (95% CI) | Mean (95% CI) | Mean (95% CI) |
| ***APOA5*** | **TT (550)** | 0.68 (0.67, 0.70) | 3.10 (3.05, 3.15) | 4.79 (4.73, 4.86) | 1.33 (1.31, 1.36) | 3.59 (3.54, 3.64) |
| **-1131C>T** | **TC/CC (102)** | 0.73 (0.69, 0.77) | 3.16 (3.03, 3.28) | 4.89 (4.74, 5.04) | 1.35 (1.30, 1.41) | 3.62 (3.50, 3.73) |
|  | **p value** | **0.048** | 0.49 | 0.24 | 0.56 | 0.74 |
|  |  |  |  |  |  |  |
| ***APOA5*** | **SS (576)** | 0.68 (0.66, 0.69) | 3.10 (3.05, 3.15) | 4.80 (4.74, 4.86) | 1.34 (1.32, 1.36) | 3.58 (3.53, 3.63) |
| **S19W** | **SW/WW (63)** | 0.74 (0.68, 0.79) | 3.12 (2.97, 3.28) | 4.83 (4.65, 5.03) | 1.33 (1.26, 1.39) | 3.63 (3.49, 3.79 |
|  | **p value** | **0.038** | 0.82 | 0.74 | 0.66 | 0.48 |
|  |  |  |  |  |  |  |
| ***APOC3*** | **S1S1 (472)** | 0.68 (0.66, 0.70) | 3.09 (3.03, 3.15) | 4.78 (4.71, 4.85) | 1.33 (1.31, 1,35) | 3.60 (3.54, 3.65) |
| **Sst1** | **S1S2/S2S2 (147)** | 0.72 (0.68, 0.77) | 3.14 (3.04, 3.25) | 4.88 (4.75, 5.00) | 1.36 (1.31, 1.40) | 3.60 (3.50, 3.70) |
|  | **p value** | **0.071** | 0.42 | 0.2 | 0.30 | 0.97 |
|  |  |  |  |  |  |  |
| ***APOC3*** | **CC (301)** | 0.68 (0.66, 0.71) | 3.07 (3.00, 3.14) | 4.75 (4.67, 4.84) | 1.33 (1.30, 1.36) | 3.58 (3.52, 3.65) |
| **-482C>T** | **CT (260)** | 0.70 (0.67, 0.72) | 3.18 (3.10, 3.26) | 4.90 (4.80, 4.99) | 1.35 (1.32, 1.38) | 3.64 (3.56, 3.71) |
|  | **TT (63)** | 0.69 (0.64, 0.74) | 3.11 (2.95, 3.26) | 4.81 (4.62, 5.00) | 1.35 (1.28, 1.42) | 3.56 (3.41, 3.71) |
|  | **p value** | 0.71 | 0.11 | 0.08 | 0.49 | 0.51 |
|  |  |  |  |  |  |  |
| ***APOC3*** | **CC (317)** | 0.68 (0.65, 0.70) | 3.08 (3.01, 3.15) | 4.76 (4.68, 4.84) | 1.32 (1.29, 1.35) | 3.60 (3.54, 3.67) |
| **1100C>T** | **CT (276)** | 0.70 (0.67, 0.78) | 3.11 (3.04, 3.19) | 4.82 (4.75, 4.91) | 1.35 (1.32, 1.38) | 3.58 (3.50, 3.65) |
|  | **TT (37)** | 0.68 (0.62, 0.75) | 2.97 (2.79, 3.17) | 4.72 (4.48, 4.97) | 1.36 (1.27, 1.45) | 3.49 (3.30, 3.69) |
|  | **p value** | 0.55 | 0.40 | 0.52 | 0.39 | 0.52 |
|  |  |  |  |  |  |  |
| ***APOA4*** | **SS (349)** | 0.68 (0.66, 0.70) | 3.09 (3.02, 3.15) | 4.79 (4.71, 4.87) | 1.33 (1.31, 1.36) | 3.58 (3.52, 3.65) |
| **S347T** | **ST (219)** | 0.71 (0.68, 0.73) | 3.09 (3.01, 3.17) | 4.79 (4.69, 4.89) | 1.33 (1.30, 1.37) | 3.60 (3.52, 3.68) |
|  | **TT (36)** | 0.67 (0.61, 0.74) | 3.25 (3.04, 3.47) | 4.98 (4.73, 5.24) | 1.37 (1.29, 1.47) | 3.62 (3.43, 3.83) |
|  | **p value** | 0.35 | 0.35 | 0.36 | 0.70 | 0.91 |
|  |  |  |  |  |  |  |
| ***LPL*** | **SS (534)** | 0.69 (0.67, 0.71) | 3.10 (3.04, 3.15) | 4.80 (4.74, 4.87) | 1.34 (1.32, 1.37) | 3.58 (3.53, 3.63) |
| **S447X** | **SX (151)** | 0.67 (0.64, 0.71) | 3.15 (3.05, 3.25) | 4.82 (4.70, 4.95) | 1.32 (1.28, 1.37) | 3.64 (3.55, 3.74) |
|  | **XX (12)** | 0.66 (0.56, 0.78) | 3.12 (2.77, 3.49) | 4.93 (4.50, 5.40) | 1.46 (1.30, 1.63) | 3.39 (3.08, 3.73) |
|  | **p value** | 0.62 | 0.69 | 0.84 | 0.25 | 0.27 |
|  |  |  |  |  |  |  |
| ***CETP*** | **B1B1 (248)** | 0.70 (0.68, 0.73) | 3.13 (3.05, 3.20) | 4.77 (4.67, 4.86) |  |  |
| **Taq1B** | **B1B2 (348)** | 0.69 (0.66, 0.71) | 3.09 (3.02, 3.16) | 4.82 (4.74, 4.90) | - | - |
|  | **B2B2 (108)** | 0.66 (0.62, 0.69) | 3.09 (2.98, 3.21) | 4.85 (4.70, 4.99) |  |  |
|  | **p value** | 0.13 | 0.78 | 0.59 |  |  |
|  |  |  |  |  |  |  |
| ***APOE*** | **E2 Carriers (33)** | 0.69 (0.67, 0.71) |  |  | 1.33 (1.30, 1.36) |  |
|  | **E3/E3 (332)** | 0.71 (0.65, 0.79) | - | - | 1.30 (1.21, 1.39) | - |
|  | **E4 Carriers (67)** | 0.71 (0.66, 0.76) |  |  | 1.29 (1.23, 1.35) |  |
|  | **p value** | 0.65 |  |  | 0.46 |  |

Tanner status was combined into one variable from the two Tanner measures, mean Tanner score.

^a^ adjusted for BMI and gender;

^b^ adjusted for height;

^c^ adjusted for height and gender;

^d^ adjusted for height, gender and BMI;

^e^ adjusted for gender, mean Tanner score and BMI;

APO – apolipoprotein, LDL-C - low-density lipoprotein cholesterol, TC - total cholesterol, HDL-C - high-density lipoprotein cholesterol, TG - total triglyceride, CI – confidence intervals, BMI- Body mass index.
