# Supplementary material for: APOE, CETP and LPL genes show strong association with lipid levels in Greek children
Source: Nutr Metab Cardiovasc Dis. 2010 Jan;20(1):26–33. doi: 10.1016/j.numecd.2009.02.005 (PMC2807029; doi:10.1016/j.numecd.2009.02.005)
Supplement: Supplementary file 3 [file mmc3.doc]

**Appendices Table 3. Univariate correlations of the variables used for principle component analysis.**

|  | **Weight** | **Waist Circum.** | **Hip Circum.** | **Triceps Skin fold** | **Subscapular Skin fold** | **Insulin Resistance** | **Insulin** | **TG** | **TC** | **HDL-C** | **LDL-C** | **TC : HDL-C** |
| --- | --- | --- | --- | --- | --- | --- | --- | --- | --- | --- | --- | --- |
| **Weight** | 1 | 0.8578 | 0.9176 | 0.7713 | 0.7879 | 0.2699 | 0.3408 | 0.3614 | 0.0570 | -0.1885 | 0.0825 | 0.27198 |
|  |  | <.0001 | <.0001 | <.0001 | <.0001 | <.0001 | <.0001 | <.0001 | 0.0974 | <.0001 | 0.0161 | <.0001 |
| **Waist** | 0.8578 | 1 | 0.8484 | 0.7621 | 0.7701 | 0.2677 | 0.3391 | 0.3537 | 0.0266 | -0.2440 | 0.0717 | 0.3069 |
| **Circum.** | <.0001 |  | <.0001 | <.0001 | <.0001 | <.0001 | <.0001 | <.0001 | 0.4360 | <.0001 | 0.0358 | <.0001 |
| **Hip** | 0.9176 | 0.8484 | 1 | 0.7682 | 0.7732 | 0.2606 | 0.3321 | 0.3345 | 0.0391 | -0.1784 | 0.0631 | 0.23314 |
| **Circum.** | <.0001 | <.0001 |  | <.0001 | <.0001 | <.0001 | <.0001 | <.0001 | 0.2544 | <.0001 | 0.0660 | <.0001 |
| **Triceps** | 0.7713 | 0.7621 | 0.7682 | 1 | 0.7335 | 0.2506 | 0.3159 | 0.2951 | 0.0674 | -0.1524 | 0.0911 | 0.22884 |
| **Skin fold** | <.0001 | <.0001 | <.0001 |  | <.0001 | <.0001 | <.0001 | <.0001 | 0.0488 | <.0001 | 0.0077 | <.0001 |
| **Subscapular** | 0.7879 | 0.7701 | 0.7732 | 0.7335 | 1 | 0.2607 | 0.3345 | 0.3837 | 0.0836 | -0.2158 | 0.1213 | 0.3248 |
| **Skin fold** | <.0001 | <.0001 | <.0001 | <.0001 |  | <.0001 | <.0001 | <.0001 | 0.0144 | <.0001 | 0.0004 | <.0001 |
| **Insulin** | 0.2699 | 0.2677 | 0.2606 | 0.2506 | 0.2607 | 1 | 0.9606 | 0.3210 | 0.0485 | -0.0916 | 0.0387 | 0.14733 |
| **Resistance** | <.0001 | <.0001 | <.0001 | <.0001 | <.0001 |  | <.0001 | <.0001 | 0.1605 | 0.008 | 0.2626 | <.0001 |
| **Insulin** | 0.3408 | 0.3391 | 0.3321 | 0.3160 | 0.3345 | 0.9606 | 1 | 0.3740 | 0.0225 | -0.1296 | 0.0144 | 0.17092 |
|  | <.0001 | <.0001 | <.0001 | <.0001 | <.0001 | <.0001 |  | <.0001 | 0.5148 | 0.0002 | 0.6769 | <.0001 |
| **TG** | 0.3614 | 0.3537 | 0.3345 | 0.2951 | 0.3837 | 0.3210 | 0.3740 | 1 | 0.2263 | -0.2441 | 0.1939 | 0.50568 |
|  | <.0001 | <.0001 | <.0001 | <.0001 | <.0001 | <.0001 | <.0001 |  | <.0001 | <.0001 | <.0001 | <.0001 |
| **TC** | 0.0569 | 0.0266 | 0.0391 | 0.0674 | 0.0836 | 0.0485 | 0.0225 | 0.2263 | 1 | 0.5347 | 0.9436 | 0.28297 |
|  | 0.0974 | 0.4360 | 0.2544 | 0.0488 | 0.0144 | 0.1605 | 0.5148 | <.0001 |  | <.0001 | <.0001 | <.0001 |
| **HDL-C** | -0.1885 | -0.2440 | -0.1784 | -0.1524 | -0.2158 | -0.0916 | -0.1296 | -0.2441 | 0.5347 | 1 | 0.2651 | -0.63229 |
|  | <.0001 | <.0001 | <.0001 | <.0001 | <.0001 | 0.008 | 0.0002 | <.0001 | <.0001 |  | <.0001 | <.0001 |
| **LDL-C** | 0.0825 | 0.0717 | 0.0631 | 0.0911 | 0.1213 | 0.0387 | 0.0144 | 0.1940 | 0.9436 | 0.2651 | 1 | 0.52232 |
|  | 0.0161 | 0.0358 | 0.0659 | 0.0077 | 0.0004 | 0.2626 | 0.6769 | <.0001 | <.0001 | <.0001 |  | <.0001 |
| **TC : HDL-C** | 0.2720 | 0.3069 | 0.2331 | 0.2288 | 0.3248 | 0.1473 | 0.1709 | 0.5057 | 0.2830 | -0.6323 | 0.5223 | 1 |
|  | <.0001 | <.0001 | <.0001 | <.0001 | <.0001 | <.0001 | <.0001 | <.0001 | <.0001 | <.0001 | <.0001 |  |

r (top) and p value (bottom) are provided for each correlation. TG - total triglyceride, TC - total cholesterol, HDL-C - high-density lipoprotein cholesterol, LDL-C - low-density lipoprotein cholesterol.
